# Assessing competency in less invasive surfactant administration: simulation-based validity evidence for the LISA-AT scores

**DOI:** 10.1038/s41390-025-03868-7

**Published:** 2025-01-18

**Authors:** Niklas Breindahl, Emma Therese Bay, Christian Heiring, Ingrid Rose MacLean-Nyegaard, Amalie Gudiksen, Andreas Frithioff, Tine Brink Henriksen, Martin Grønnebæk Tolsgaard, Lise Aunsholt

**Affiliations:** 1 Department of Neonatal and Pediatric Intensive Care, Copenhagen University Hospital, Copenhagen, Denmark; 2 Faculty of Health and Medical Sciences, University of Copenhagen, Copenhagen, Denmark; 3 Prehospital Center Region Zealand, Næstved, Denmark; 4 Copenhagen Hearing and Balance Center, Department of otorhinolaryngology, Copenhagen, Denmark; 5 Department of Paediatrics and Adolescent Medicine, Aarhus University Hospital, Aarhus, Denmark; 6 Perinatal Research Unit, Department of Clinical Medicine, Aarhus University, Aarhus, Denmark; 7 Copenhagen Academy for Medical Education and Simulation (CAMES), Copenhagen University Hospital, Copenhagen, Denmark; 8 Department of Obstetrics, Copenhagen University Hospital Rigshospitalet, Copenhagen, Denmark

## Abstract

**Background:**

The Less Invasive Surfactant Administration Assessment Tool (LISA-AT) was developed to support operator training and competence assessment. This study aimed to gather validity evidence in the simulated setting to support using the LISA-AT scores.

**Methods:**

Validity evidence was gathered using the Messick framework. The lowest quartile (Q1) for the median of the experts’ LISA-AT scores defined the minimum passing score.

**Results:**

Ten experts and 23 novices were enrolled in this study. Eight of the original 15 LISA-AT metrics effectively discriminated between novices and experts and demonstrated high test-retest reliability (Spearman’s rho = 0.87), high internal consistency, and good inter-rater reliability (Cronbach’s alpha = 0.88 and 0.82, respectively). The LISA-AT discriminated between novices’ and experts’ first two attempts with median [IQR] scores of 29 [26–32] vs 39 [39–40]). The minimum passing score was defined as 39/40 points, and the novices used a median [IQR] of 6 [5–7] attempts, ranging from 4 to 9 attempts, to reach this score. Compared with the expert group, the novices’ laryngoscopy skills and time remained significantly different even after attaining the minimum passing score.

**Conclusion:**

We found strong validity evidence to support using the LISA-AT scores to train new LISA operators to the minimum passing score to ensure competence in the simulated setting.

**Impact:**

- This study showed robust validity evidence for using the Less Invasive Surfactant Administration Assessment Tool (LISA-AT) to train LISA novices with a high test-retest reliability, high internal consistency, and good inter-rater reliability.
- Eight of the original 15 LISA-AT metrics effectively discriminated between novices and experts, with a minimum passing score of 39/40 points, corresponding to the lowest quartile (Q1) of the experts’ performances. Novices typically needed six attempts in the simulated setting, ranging from four to nine. However, their laryngoscopy skills and duration remained significantly different from experts.
- Using the LISA-AT score can ensure competence in the simulated setting before advancing to supervised clinical procedures.

## Introduction

Less Invasive Surfactant Administration (LISA) is a method of surfactant administration to preterm infants with increasing oxygen demands despite optimised non-invasive ventilation. By supplying surfactant, LISA aims to prevent alveolar collapse while avoiding endotracheal intubation and invasive ventilation before, during, and after surfactant administration^1–3^. According to the European Consensus Guidelines on Management of RDS, LISA is the preferred method of surfactant administration in spontaneously breathing preterm infants^1,4^ and LISA is becoming more widely used in neonatal intensive care units (NICUs) worldwide^5–8^.

LISA is a complex procedure characterised by surfactant administration through a thin endotracheal catheter using laryngoscopy while supporting spontaneous breathing by applying non-invasive respiratory support^2,3,9,10^. Effective and safe performance of LISA requires a clinician with experience in the LISA procedure, rescue intubation, and non-invasive respiratory support^9,11,12^. However, this is often difficult to establish due to a low monthly incidence of LISA procedures per operator even in moderately sized NICUs^12^. Simulation-based training is the recommended method for acquiring competence in LISA because it can be achieved in a safe, controlled environment without harming the patient^13,14^. A review from 2018 showed that 72% of physicians using LISA in the United States had no formal training on a mannequin before performing the procedure on infants^8^.

The LISA curriculum (LISA-CUR) and the LISA assessment tool (LISA-AT) were established by international consensus in 2023 to support training and assessment of operator competence^12^. However, there was no validity evidence to support the use of the LISA-AT scores to evaluate trainee competence.

This study aimed to gather validity evidence in the simulated setting to support the LISA-AT scores, establish a minimum passing score, and explore learning curves to estimate the number of attempts needed for novices to reach a predefined mastery level in a simulated setting.

## Methods

### Study design

This study was conducted from March to November 2023 at the Department of Neonatal and Paediatric Intensive Care at Aarhus University Hospital and Copenhagen University Hospital (Rigshospitalet), Denmark. We assessed the validity evidence to support the LISA-AT scores using Messick’s framework recommended by the American Educational Research Association in their Standards for Education and Psychological Testing^15–17^. This framework includes five different sources of validity evidence: (1) content evidence, (2) response process, (3) internal structure, (4) relations to other variables, and (5) consequences of testing^18^. Finally, the micro-costing approach^19^ was used to evaluate the cost of training one novice to the minimum passing score. We included four steps: (1) specification of resources used (time, materials, and equipment), (2) determination of the quantities of resources, (3) determination of the unit costs, and (4) multiplication of the quantities and the unit costs^20,21^.

### Development of the LISA assessment tool (LISA-AT)

In 2023, 153 LISA experts from 14 countries participated in a modified three-round Delphi process to establish international expert consensus for the LISA curriculum (LISA-CUR) and the LISA assessment tool (LISA-AT, available in the Online Supplement, Appendix A). The original LISA-AT is described previously (see Content evidence)^12^.

### Participants

Novices and experts were included in a 2:1 ratio as a convenience sample. The novices included final-semester medical students and young medical doctors without clinical experience in endotracheal intubation or LISA. Medical students were recruited during their clinical rotation in paediatrics, and medical doctors were recruited from the paediatric departments in the Capital Region and Region Zealand of Denmark. The experts included senior staff neonatologists experienced in endotracheal intubations and the LISA procedure. Five experts were enrolled from both NICUs. All participants gave consent and received no compensation for participation.

### Simulation equipment

**Fig. 1:**
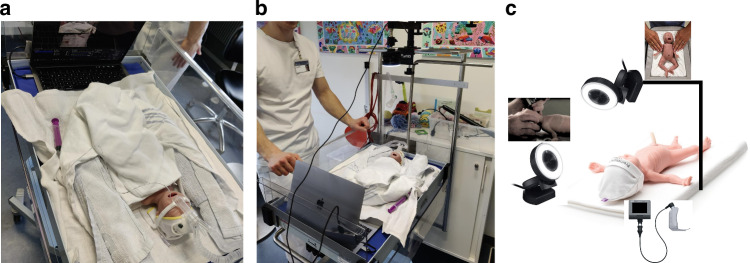
Simulation setting and camera output. **a**,**b** The simulation setting. **c** The simulation setup used 3 external cameras, including (1) a sagittal view of the simulator’s head and neck, (2) a bird’s-eye view of the entire simulator, and (3) the operator’s point of view (POV) through the video laryngoscope.

Equipment used was the C-MAC® video-laryngoscope HD series, a Miller blade size 0 (Karl Storz™, Germany)^22^, the Surfcath (Vygon™, United Kingdom)^23^, and the Premature Anne mannequin (Laerdal Medical™, Norway, Stavanger) simulating a preterm infant born at 25 weeks and birth weight 750 grams^24^. The scenario was performed in a simulated single-family room (Fig. 1a,b). Videos were obtained from the video-laryngoscope and from two static video cameras: one placed above the mannequin and one placed next to the mannequin’s head to visualise the head positioning (Fig. 1c). The recordings did not include footage of the participant’s faces.

### Simulation setting

**Fig. 2:**
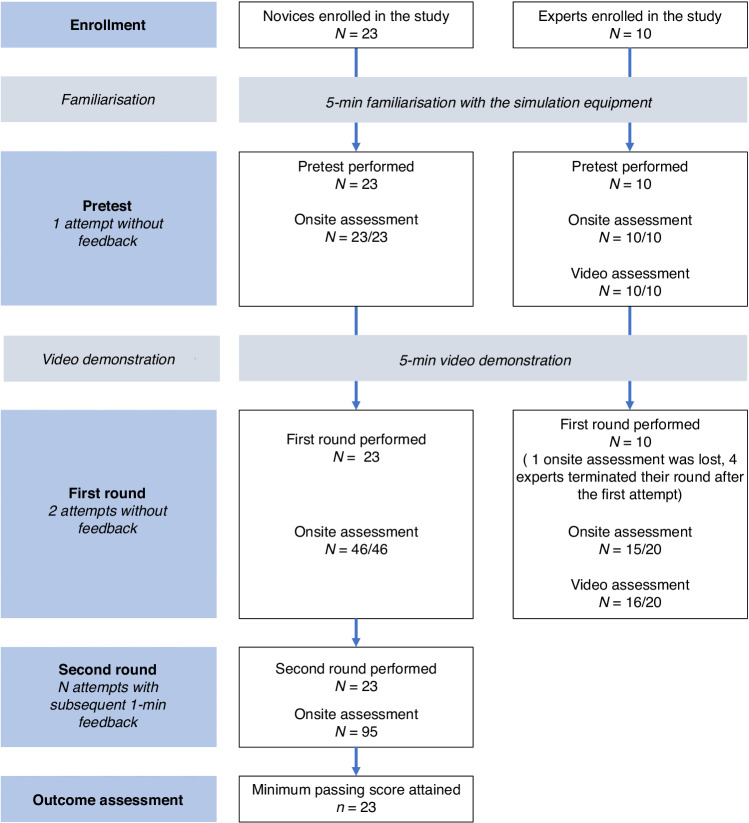
Study flowchart. Ten experts and 23 novices were enrolled in this study. Both novices and experts were allowed 5 min of familiarisation with the equipment before completing the pretest. The first round consisted of two consecutive attempts without feedback. The novices continued training in the second round until they achieved the minimum passing score in two consecutive attempts. One minute of feedback was allowed after each attempt.

Figure 2 displays the simulation and data flow. All participants were allowed five minutes to familiarise themselves with the simulation equipment before beginning the first simulation scenario. All experts and novices were given a short test introduction telling them to behave similarly to what they would in real life to increase the psychological realism (Online Supplement, Appendix B). The participants performed a practical pretest and watched a demonstration video. The pretest was identical to the subsequent scenarios. The demonstration video highlighted key actions and provided a step-by-step visual guide on performing the LISA procedure, focusing on the equipment and the technique. Afterwards, the participant performed the first round (two LISA scenarios without feedback). LISA-AT metrics that did not discriminate between the novices and experts in the first round were removed. The minimum passing score was established based on the experts’ performances in the first round (see Statistical analysis). Only the novices continued practising in the second round until they attained the minimum passing score in two consecutive attempts. After each attempt in the second round, the novices received one minute of feedback on how to improve their LISA-AT score. The novices were not informed of their score after each attempt but received feedback on the metrics with scores lower than the maximum score of five points. A training session had a maximum duration of 90 min to prevent participant fatigue.

Two investigators were present during data collection. Investigator A was responsible for the ratings using the LISA-AT (Online Supplement, Appendix A)^12^. Investigator B played the role of two neonatal nurses assisting during all performances but was unable to perform any actions without being instructed and did not offer any advice to the participant during the scenario (i.e., if Investigator B was instructed to perform non-pharmacological measures, Investigator B would reply: “*How should I do that?*”). Managing complications was a part of the original LISA-AT^12^ and was defined specifically for this study as bradycardia, desaturation, and surfactant reflux, played in consecutive order, one for each scenario. Otherwise, the scenarios were identical. These complications aimed to increase the fidelity of the simulation to better mimic the clinical environment (Online Supplement, Appendix C). Investigator B, acting as the neonatal nurse, was instructed to incorporate the complications during the surfactant administration by verbalizing the problem (e.g., bradycardia, desaturation, or surfactant reflux).

### Rater training

Four investigators, including two onsite investigators (a physician and a medical student) and two video investigators (senior staff neonatologists), assessed four participants not included in the study (three novices and one expert), followed by a comparison of scores. Based on the group discussions, the assessment guide was reworded while the content was unchanged (Online Supplement, Appendix C).

The video ratings were considered the gold standard but resource-demanding and impractical as they could not provide immediate feedback to the participants. On the contrary, using non-specialist, trained investigators as onsite raters was considered more feasible to provide immediate feedback at a lower cost. Therefore, this study compared the trained investigators’ onsite and senior staff neonatologists’ video ratings of the experts’ performances in the first round. If no differences could be detected, trained investigators would be used to rate the novices’ performances onsite in the second round (Fig. 2).

### Data collection

The participants provided their baseline characteristics, including their level of experience with simulation and the LISA procedure. All LISA-AT forms were filled out on paper and entered into the Research Electronic Data Capture (REDCap) system for data collection^25,26^. All videos were processed and merged using OBS Studio 28.0.1 and safely stored on a secure drive.

### Ethical considerations

All participants provided written informed consent, including approval of video recordings at the first simulation session before data collection. Participation was anonymous, and all information was handled confidentially. This study was exempt from ethical approval according to Danish legislation (protocol number 22031294). Data management and processing were approved (PRIVACY journal number: P-2022-318).

### Statistical analyses

R Studio version 3.5.3 was used for statistical analysis^27^. Baseline characteristics were presented separately for each group and the total study population. Categorical variables were presented as frequencies (counts and percentages) and numerical variables as medians with interquartile ranges (IQR) due to non-normality (inspected using histograms and Q-Q-plots). Analyses were performed using the Chi-squared test, Mann–Whitney U test, Kruskal-Wallis test, and Wilcoxon sign test, as appropriate. *P*-values were adjusted for multiple testing using the Hochberg method^28^.

The internal structure was assessed in terms of the reliability of the LISA-AT over time (test-retest reliability), across items (internal consistency), and different raters (inter-rater reliability).

The test-retest reliability reflects the variation in measurements taken by an instrument on the same subject under the same conditions. It was calculated by comparing the participants’ LISA-AT scores in the first two attempts using Spearman’s correlation coefficient and Intraclass Correlation Coefficients (ICC, model = two-way random-effects, type = agreement, unit = single)^29^. A correlation of >0.80 indicated good reliability^29^.

The internal consistency for all LISA-AT items was calculated by comparing the participants’ LISA-AT scores in the first round using Cronbach’s Alpha.

The inter-rater reliability was calculated using ICC (model = two-way random effects, type = agreement, unit = single)^29^.

We applied generalizability (*G*) theory to assess the overall reliability of the LISA-AT scores in the second round. To obtain a fully-crossed design, only the first 6 performances were included in the *G* study. This included two facets: Participant and Performance number, as the performances in the second round were only assessed by one rater. A *G*-coefficient >0.80 was considered appropriate for high-stake assessment^30,31^. We performed subsequent decision (D) studies to estimate the optimal number of performances required to reach a reliable assessment.

The consequences of testing were determined by a minimum passing score using the lowest quartile (Q1) of the LISA-AT scores for the experts.

## Results

### Participant demographics

Ten experts and 23 novices were enrolled in this study. Table 1 shows the baseline characteristics. The experts had performed a median [IQR] of 11 [10–14] unsupervised LISA procedures, ranging from seven to 30 procedures before enrollment. Figure 2 illustrates the study flowchart, including the number of performances during the rounds. Table 2 summarises the five sources of validity evidence.

**Table 1:** Participants’ baseline characteristics.

	Novices (*N* = 23)	Experts (*N* = 10)	Total (*N* = 33)
Sex, *n* (%)			
Female	15 (65)	4 (40)	19 (58)
Male	8 (35)	6 (60)	14 (42)
Age, years, median [IQR]	31 [28–37]	48 [46–50]	35 [29–45]
Clinical experience, years, median [IQR]	3 [2–7]	20 [18–20]	5 [2–18]
LISA procedures, median [IQR], range			
Primary operator, simulated	NA	14 [5–20], 0–50	NA
Primary operator, supervised	NA	2 [2], 0–5	NA
Primary operator, unsupervised	NA	11 [10–14], 7–30	NA
Primary operator, within 30 days	NA	1 [0–1], 0–2	NA

Baseline characteristics of the novices and the experts. *IQR* interquartile ranges, *NA* not applicable.

**Table 2:** Sources of validity evidence to support the interpretation of LISA-AT scores using the Messick framework.

Sources of evidence for validity	Description of source	Validity evidence for the LISA-AT
Content	The LISA-AT should measure the intended goals and objectives	The LISA-AT content, including response anchors, was developed by 153 LISA experts from 14 countries.
Response process	The integrity of data should always be maintained. Test administration should be controlled or standardised at the highest level	The LISA-AT scores of the experts’ performances (the first round) showed no significant differences between onsite and video ratings (*p* = 0.35). The novices were trained according to a prespecified protocol by trained raters onsite.
Internal structure	The LISA-AT should be reliable over time (test-retest), across items (internal consistency), and different raters (inter-rater).	The LISA-AT had a high test-retest reliability (Spearman’s rho = 0.87 and ICC [IQR] = 0.81 [0.63-0.90]), high internal consistency (Cronbach’s alpha [IQR] = 0.88 [0.84–0.91]), and moderate-good inter-rater reliability (Cronbach’s alpha [IQR] = 0.82 [0.68-0.97] and ICC [IQR] = 0.71 [0.38–0.88]). The global rating scale was highly correlated with the total modified LISA-AT score for pairwise comparisons (*p* < 0.001). Overall *G*-coefficient was 0.73. The largest contributor to the variance was the number of procedures performed by the participant (53%). Subsequent decision (D) studies established that 9 procedures assessed by one rater would be sufficient for obtaining a *G*-coefficient >0.80.
Relations to other variables	Assessment scores should correlate with known measures of competence	Seven LISA-AT metrics did not discriminate significantly between the novices and experts and were removed to form the modified LISA-AT. The novices had significantly lower modified LISA-AT scores compared to the experts in the first round (median [IQR]: 29 [26–32] vs 39 [39-40].
Consequences	Consequences of testing are supported by the pass/fail standard that is set	The lowest quartile (Q1) for the median modified LISA-AT score in the experts’ first round was used to set the minimum passing score at 39 points (higher than the intercept between novices and experts, which was 36 points.

The five sources of validity evidence according to the Messick framework. *LISA* less invasive surfactant administration, *LISA-AT* LISA assessment tool, *ICC* intraclass correlation coefficient, *IQR* interquartile ranges.

### Content evidence

The LISA-AT was developed in a separate study and contained 15 metrics rated on a Likert scale of one to five with a range from 15 to 75 points, and a global rating scale (pass/borderline/fail)^12^. Descriptive anchors were provided for scores one, three, and five to guide the investigators (Online Supplement, Appendix A). The duration of laryngoscopy was defined to assess the time from introducing the laryngoscope blade for the first time to removing the blade from the simulator’s mouth after a successful LISA procedure.

### Response process

The response process is described under “Data collection” in the Methods section. The median [IQR] LISA-AT score in the first round showed no significant differences between onsite and video ratings of the experts’ performances (73 [73] vs 74 [72–75], *p* = 0.35 [Online Supplement, Appendix D]). Therefore, trained investigators were used as onsite raters to train the novices.

### Internal structure

The internal structure of the LISA-AT was assessed during the first round.

The LISA-AT showed good reliability over time (test-retest reliability), with a Spearman’s rho = 0.87 [Online Supplement, Appendix E]), and ICC [IQR] = 0.81 [0.63–0.90].

The LISA-AT showed good reliability across items (internal consistency) with a Cronbach’s alpha [IQR] = 0.88 [0.84–0.91]. The LISA-AT showed moderate to good reliability across different investigators (inter-rater reliability) with a Cronbach’s alpha [IQR] = 0.82 [0.68-0.97] and ICC [IQR] = 0.71 [0.38–0.88].

The overall G-coefficient was 0.73 indicating a moderate to good level of reliability of the LISA-AT scores in the second round. The largest contributor to the variance was the number of performances (53%). Subsequent decision (D) studies established that 9 performances assessed by one rater would be sufficient for obtaining a *G*-coefficient >0.80 to reduce the rate of measurement error or other unmeasured sources of variability.

There was an overall significant correlation between the global rating scale (pass/borderline/fail) and the total LISA-AT score. The pairwise comparisons showed significantly different distributions of the total LISA-AT scores within each category of the global rating scale (Online Supplement, Appendix F).

### Relations to other variables

Table 3 shows the novices’ and experts’ performances in the first round for each LISA-AT metric. When adjusting for multiple testing using the Hochberg method^28^, seven metrics (“*Pharmacological interventions*”, “*Non-pharmacological interventions*”, “*Positioning*”, “*Team briefing*”, “*Catheterisation*”, “*Non-technical skills*”, and “*Number of attempts*”) did not discriminate significantly between the novices and experts and were excluded from the LISA-AT (Online Supplement, Appendix A). The total LISA-AT scores in this study were calculated as the sum of the remaining eight metrics ranging from 8-40 points (“*Monitoring*”, “*Equipment preparation*”, “*Laryngoscopy*”, “*Surfactant administration*”, “*Complications*”, “*Ventilation*”, “*Adherence to algorithm / Time factor*”, “*Handling of the infant*”).

**Table 3:** The LISA-AT metrics’ ability to discriminate between the novices’ and experts’ performance in the first round.

LISA-AT metrics	Novices *N* = 46	Experts *N* = 15	*P*-value unadjusted^α^	*P*-value adjusted^β^
Pre-procedure, median [IQR]				
Monitoring	3 [2–4]	5 [5]	<0.001*	0.001*
Equipment preparation	4 [3–5]	5 [5]	<0.001*	0.003*
Pharmacological interventions	5 [3–5]	5 [5]	0.023*	0.092
Non-pharmacological interventions	4 [4,5]	4 [4,5]	0.825	0.825
Positioning	5 [3–5]	5 [4,5]	0.287	0.575
Team-briefing/Team resource management	5 [4,5]	5 [5]	0.123	0.368
Procedure, median [IQR]				
Laryngoscopy	3 [2,3]	5 [5]	<0.001*	0.001*
Catheterisation	4 [3–5]	5 [5]	0.008*	0.056
Surfactant administration	4 [4,5]	5 [5]	0.005*	0.039*
Complications	3 [2–5]	5 [5]	<0.001*	0.002*
Ventilation	4 [3–5]	5 [5]	0.002*	0.017*
Non-technical skills, median [IQR]				
Non-technical skills	5 [4,5]	5 [5]	0.022*	0.092
Overall, median [IQR]				
Number of attempts	5 [4,5]	5 [5]	0.012*	0.075
Adherence to algorithm / Time factor	4 [3–5]	5 [4,5]	0.001*	0.014*
Handling of the infant	3 [3,4]	5 [5]	<0.001*	0.001*

^α^All analyses were performed using the Chi-squared test.^β^adjusted for multiple testing using the Hochberg method.*significant *p*-value < 0.05. *n* count the number of attempts assessed by onsite raters. A total of 23 novices and 10 experts performed two attempts, but one of the experts’ attempts in the first round went missing and four experts terminated their simulation training after the first attempt. Seven metrics did not discriminate significantly between the novices and the experts when adjusting for multiple testing using the Hochberg method. All significant metrics were included in the modified LISA-AT score. *IQR* interquartile range, *LISA-AT* Less invasive surfactant administration assessment tool.

### Consequences of testing

The novices had significantly lower LISA-AT scores compared to the experts in the first round (median [IQR]: 29 [26–32] vs 39 [39,40], *p* < 0.001). The minimum passing score was set at a LISA-AT score of 39 points, corresponding to the lowest quartile (Q1) of the experts’ performances. Setting the minimum passing score of 39 points resulted in zero false positives (passing novices) and four false negatives (non-passing experts).

### Learning curves

**Fig. 3:**
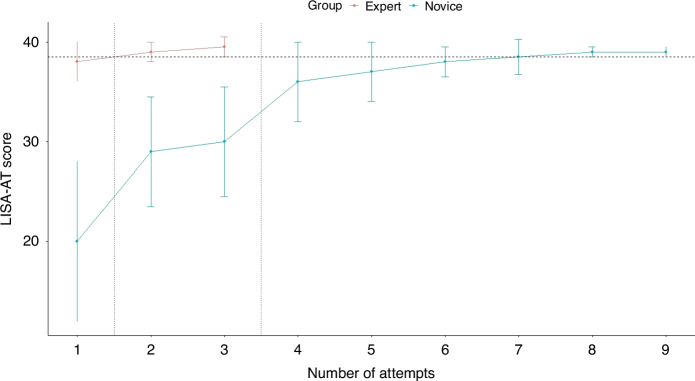
Learning curves for the participants through the pretest, first, and second rounds. The vertical lines indicate the transition from the pretest to the first round (after the demonstration video, without feedback) to the second round (with feedback). The horizontal line indicates the minimum passing score of 39 points. The median [IQR] number of attempts required for the novices to reach the minimum passing score in the simulation setting was 6 [5–7], ranging from 4 to 9. Each attempt took approximately 10 min, and all novices achieved the minimum passing score within the time limit of 90 min.

Figure 3 displays the learning curves for the novices. The median [IQR] number of attempts required for the novices to reach the minimum passing score in the simulation setting was 6 [5–7], ranging from 4 to 9, which was possible within one training session of 90 min. The duration of laryngoscopy significantly decreased from the pretest to the first round (Online Supplement, Appendix G). There was no improvement in the duration of laryngoscopy during the second round with feedback, which remained different from the experts’ performances (median [IQR] for the novices was 36 s [27–47] compared to 20 s [15–26] for the experts, *p* < 0.001, Table 4). The novices’ laryngoscopy skills significantly improved between the pretest and the subsequent rounds (Online Supplement, Appendix H). However, they remained significantly different from the experts’ performances (*p* < 0.001, Table 4).

**Table 4:** Comparison of the novices’ and experts’ performances after the novices achieved the minimum passing score in the second round.

LISA-AT metric	Novices *N* = 95	Experts *N* = 15	*P*-value unadjusted^α^	*P*-value adjusted^β^
Pre-procedure, median [IQR]				
Monitoring	5 [5]	5 [5]	0.259	0.783
Equipment preparation	5 [5]	5 [5]	0.418	0.783
Procedure, median [IQR]				
Laryngoscopy	4 [3–5]	5 [5]	<0.001*	0.001*
Surfactant administration	5 [5]	5 [5]	0.783	0.783
Complications	5 [5]	5 [5]	0.319	0.783
Ventilation	5 [5]	5 [5]	0.435	0.783
Overall, median [IQR]				
Adherence to algorithm/Time factor	5 [4,5]	5 [5]	0.329	0.783
Handling of the infant	5 [4,5]	5 [5]	0.014*	0.151
Laryngoscopy time, median [IQR]	36 [27–47]	20 [15–26]	0.001*	0.001*

^α^All analyses were performed using the Chi-squared test except for laryngoscopy time analysed using the Mann–Whitney U test.^β^adjusted for multiple testing using the Hochberg method.*significant *p*-value < 0.05. *n* count the number of attempts in the novices’ second round and the experts’ first round assessed by onsite raters. Two metrics discriminated significantly between the novices and the experts even after the novices achieved the minimum passing score “*Laryngoscopy”* and “*Laryngoscopy time”*. *IQR* interquartile range, *LISA-AT* less invasive surfactant administration assessment tool.

### Evaluation of cost

The simulator cost was USD 3149 (excluding VAT)^32^. Assuming straight-line depreciation over 5 years and 50 days of use per year (7 h per day), the hourly price was determined at USD 2^21^. We were able to perform up to nine attempts within the time restriction of 90 min (one attempt per 10 min). Based on the micro-costing procedure, the direct costs of onsite LISA simulation training with basic simulators using trained investigators as onsite raters were USD 68, ranging from USD 45–102 per novice to achieve the minimum passing score (Table 5). One-way sensitivity analysis identified the most important variables as the raters’ salary. If senior staff neonatologists were employed as raters, the unit costs would increase from USD 22 to USD 67, increasing the median (range) expenditure for raters from USD 44 (29–66) to USD 134 (90–201). Other considerations included training paediatric residents or interdisciplinary team-training with higher opportunity costs than a single novice, assuming off-site simulation with increased facility costs^21^, or using a more advanced simulator^33^.

**Table 5:** Direct cost of simulation-based LISA training of one novice.

Expense	Unit costs [USD]	Quantities of resources	Total [USD]
Primary analysis, median (range)			
Trained investigators as onsite raters	22	2 × [time_MPS_]	44 (29–66)
Novices as trainees (opportunity costs)	22	[time_MPS_]	22 (15–33)
Depreciation of simulator	2	[time_MPS_]	2 (1–3)
Total [USD]			68 (45–102)
One-way sensitivity analysis, median (range)			
Assume consultant-level neonatologists as onsite raters	67	2 × [time_MPS_]	134 (90–201)
Assume paediatric residents as trainees (opportunity costs)	35	[time_MPS_]	35 (23–53)
Assume team training (one paediatric resident [PR] and two neonatal nurses [NN])	PR: 35 NN: 28	1PR × [time_MPS_] 2NN × [time_MPS_]	91 (61–137)
Assume off-site simulation	27	[time_MPS_]	27 (18–41)
Assume more advanced simulator	32	[time_MPS_]	32 (21–48)

The calculations did not consider the cost of developing the LISA-AT, as this tool was already developed, and acquiring a video-laryngoscope and other procedure-related utensils, as the department already had these in custody. Two simulator instructors were required for LISA training (one assessor and one acting as the assisting nurses). We anticipated identical hourly expenses between the trained investigator’s salary and the novice’s opportunity costs. The novices in this study were unpaid, but they may have used their time for paid work elsewhere if they had not participated in this study. Therefore, it is essential to acknowledge opportunity costs. The quantities were calculated from timeMPS (the time required to reach the minimum passing score), as one attempt took 10 min. The median (range) number of attempts to achieve the minimum passing score was 6 (4–9) attempts, corresponding to 60 (40–90) min. Assuming depreciation over 5 years and 50 days of use per year (7 h per day), the hourly price was determined at USD 2 with a basic simulator and 32 with an advanced simulator. We estimated the hourly expenses of consultants, residents, and nurses based on Danish salary agreements. Trained investigators and facility costs were based on the article by Post et al. (2023) using the same Danish setting. All cost estimates are subject to assumptions and rounded up to integers.*LISA* less invasive surfactant administration, *USD* United States dollar, *NN* neonatal nurse, *PR* paediatric resident, *time*_MPS_ the time required to reach the minimum passing score.

## Discussion

This study found robust validity evidence for using the LISA-AT scores to train new LISA operators using the Messick framework. Using the LISA-AT scores can ensure competence in the simulated setting before the operator is promoted to training in the clinical setting under the supervision of an experienced LISA operator. The LISA-AT can be used to train novices to a predefined minimum passing score at low expenses. LISA-AT scores can provide ongoing assessments and feedback during each attempt (formative assessment) and for a final assessment to measure the learners’ competency and determine when the minimum passing score has been achieved (summative assessment).

The safe performance of LISA requires an experienced interdisciplinary team^1^. However, the team’s experience with LISA may be limited due to the relatively small number of infants eligible for LISA^12^. Standardised simulation-based LISA training has been recommended in the European Technical Skills Curriculum in Neonatology^14^ to improve LISA success rates^13,34,35^.

A previous study showed improved success rates of LISA in the clinical setting following a standardised approach to LISA training involving an educational film, checklists, pocket cards, and team briefings^35^, but the medical staff did not train until reaching a predefined minimum passing score. Setting a minimum passing score is an essential asset of an assessment tool^15,36,37^, which is required in competency-based learning for procedural assessment to guarantee that all trainees possess the essential skills required for safe practice^38,39^. In this study, there were no consequences of time-based education, as all novices achieved the minimum passing score within the time limit of 90 min.

The need for continued training is highlighted by the significant differences in the laryngoscopy skills and duration of laryngoscopy time observed between the experts and the novices, even after the novices achieved the minimum passing score. Similar to other clinical skills rarely performed in the clinical setting, periodic re-training may be essential. However, this study was not designed to evaluate skill retention or the transferability of skills from the simulated setting to clinical practice. Seven metrics did not discriminate between the novices and experts in the first round. This is likely because nurses perform some of the metrics (an onsite investigator in this study), and the participants were asked to mention the interventions, not perform them (e.g., “*Non-pharmacological interventions*”). Therefore, an acceptable score was too easy to achieve. Other metrics may have been challenging to observe and evaluate in the simulation setting by the onsite investigators (e.g., “*Positioning*”). Future studies could explore the integration of sensor technology to enhance the extent and precision of skill evaluation; however, this study prioritised using standard training manikins widely available in clinical settings to ensure the results would be broadly applicable. Some metrics were easy to remember and learn just by watching the demonstration video (e.g., “*Team-briefing*”). However, these metrics are still crucial for the LISA procedure’s success and should be addressed in the training of the clinical staff. Preferably, this training should involve both nurses and doctors on an interdisciplinary team to allow for the safe practice of both procedural skills, social and cognitive skills (i.e., non-technical skills and team-briefing), and the non-pharmacological approach to promote the safety and success of the LISA procedure^40^.

A recent study by Rostoker et al.^41^ developed a rating scale to teach LISA in simulation using the same sources of validity evidence^42^ and a modified Delphi process similar to Breindahl et al.^12^. However, Rostoker et al. only included 12 neonatologists from two countries (France and Belgium), and Breindahl et al. included 153 LISA experts from 14 countries. The final rating scale by Rostoker et al. was composed of 25 metrics in 8 categories with three response anchors (“*not completed*”, “*partially completed*”, or “*perfectly completed*”). The LISA-AT was composed of 15 metrics with five response anchors. Like Rostoker’s rating scale, the LISA-AT considered the lack of consistency regarding premedication and could be used to train operators across units. While the Rostoker’s rating scale focused exclusively on the operator’s technical skills, the LISA-AT emphasised the importance of non-pharmacological interventions and several social and cognitive skills (i.e., “*team briefing*” and “*non-technical skills*”), which are integral elements of a successful LISA and may be suitable for interdisciplinary team training. Rostoker et al. included six experts with an average score of 73.6% and suggested that novices achieving the average score (i.e., a minimum of 36.8/50 points) could perform the procedure^41^. However, a score of 37/50 can be achieved even though 6/25 metrics are “*not completed*” (scoring 0) or 13/25 metrics are “*partially completed*” (scoring 1). To achieve the minimum passing score on the LISA-AT and be promoted to training in the clinical setting under the supervision of an experienced LISA operator, the participant must achieve the highest score of five points on all metrics except for two metrics, scoring four points, but if only one metric scores three points the minimum passing score cannot be achieved. Thus, using the LISA-AT ensures a high level of competence. However, both tools still need to be translated into clinical performance.

This study has some limitations. It was impossible to blind the onsite investigators, thereby introducing experimenter bias, where the investigator may behave differently with the experts and novices, which can impact the assessments. Experimenter bias was minimised through a standardised assessment protocol (Online Supplement, Appendix C) and could have been minimised further by adding a second rater in the second round. Blinding is important to increase objectivity and reduce bias and could have been achieved through video assessment. However, the ability to provide feedback after each attempt would then have been impossible. Additionally, the total score following each attempt was critical in determining when to conclude the second training round, making live assessment essential to the study’s design. The small sample size may have been a limitation to the internal validity, but it was evident from the pretest assessments that the experts and novices represented two separate samples. Including only Danish participants may have been a threat to the external validity. However, we accounted for this by including participants from multiple institutions. The maximum training time of 90 min was chosen to prevent fatigue among the participants, but adding a time restriction may have impacted the assessments, as the investigators may have been more likely to give a higher score towards the end to complete the data collection for that participant without needing to schedule a second training. Finally, this study only focused on collecting validity evidence for the LISA-AT scores by assessing the ‘neonatologist’ performance and therefore did not include neonatal nurses. However, the LISA procedure requires a proficient interdisciplinary team to be successful in a clinical environment, and a multidisciplinary team training approach should be considered for future LISA simulation-based training. This should include the assessment of skills that rely on teamwork like non-pharmacological interventions, team briefing, and non-technical skills.

This study has several strengths. Using the Messick framework as a systematic approach to collect different aspects of validity evidence strengthens the overall validity argument for the LISA-AT. The test-retest reliability in the first round without feedback was high, indicating a limited testing effect, where the participant becomes better from just taking the test. Hence, the improvements in the novices’ performances during the second round can be attributed to the feedback provided by the investigators, meaning that the LISA-AT can be used for formative assessment to identify procedural deficiencies and provide structured feedback to achieve the learning objectives. The effect of maturation (learning over time from other reasons) was limited, as all novices achieved the minimum passing score within 90 min, limiting the drop-out rate and related attrition bias. Diffusion in terms of crosstalk or observation between participants was eliminated as the participants were enrolled individually on different days. Finally, this study demonstrated that simulation-based LISA training can be conducted at low costs using onsite trained raters.

## Conclusion

Robust validity evidence using the Messick framework supported using LISA-AT scores to train new LISA operators. The LISA-AT can be used for formative and summative assessment in simulation-based LISA training. The high minimum passing score emphasises the need for novices to train in a simulation-based environment before being promoted to training in the clinical setting under the supervision of an experienced LISA operator.

## Supplementary information

Supplement_Appandix_A, clean version

Supplement_Appendix_B

Supplement_Appandix_C

Supplement_Appendix_D

Supplement_Appendix_E

Supplement_Appendix_F

Supplement_Appendix_G

Supplement_Appendix_H

## Data Availability

The datasets generated and analysed for this study are available from the corresponding author upon reasonable request.
