# Supplementary material for: Assessing competency in less invasive surfactant administration: simulation-based validity evidence for the LISA-AT scores
Source: Pediatr Res. 2025 Jan 18;98(3):876–84. doi: 10.1038/s41390-025-03868-7 (PMC12507647; doi:10.1038/s41390-025-03868-7)
Supplement: Supplementary file 1 — Supplement_Appandix_A, clean version [file 41390_2025_3868_MOESM1_ESM.pdf]

Appendix A: The LISA assessment tool (LISA-AT) with response anchors

**LISA-AT metric**	**Response anchors**				
Pre-procedure	*1*	*2*	*3*	*4*	*5*
Monitoring*<br>*Ensure appropriate monitoring of vital signs before and during the LISA procedure.*	Fails to ensure monitoring.		Competent but not consistent monitoring of vital signs.		Excellent monitoring of vital signs before and during the procedure.
Equipment preparation*<br>*Ensure all equipment used in the LISA procedure is functioning.*	Fails to check equipment / checks equipment incorrectly.		Competent but not consistent check of the equipment.		Excellent check of all the equipment.
Pharmacological interventions<br>*Check drugs and doses that may be used as part of the LISA procedure (including antidote if opioid is being used).*	Fails to check drugs.		Competent but not consistent check of drugs.		Excellent check of all drugs and doses.
Non-pharmacological interventions<br>*Ensure non-pharmacological precautions on pain and stress management.*	Displays poor knowledge on non-pharmacological precautions.		Displays some knowledge of non-pharmacological precautions		Displays ample knowledge of non-pharmacological precautions.
Positioning<br>*Position the patient optimally and maintain position during the LISA procedure.*	Fails to position the patient.		Competent but not consistent position of the patient throughout the procedure.		Excellent position of the patient during the procedure.
Team-briefing / Team resource management<br>*Perform a team briefing before the LISA procedure including task assignment, outlining potential problems etc.*	Fails to perform team briefing.		Performs team briefing with some experience.		Excellent team briefing.
Procedure	*1*	*2*	*3*	*4*	*5*
Laryngoscopy*<br>*Competent and non-injurious handling of the laryngoscope and good visualisation of the vocal cords including sufficient overview of the airway.*	Fails to handle the laryngoscope / Injurious handling of the laryngoscope / Fails to visualise the vocal cords.		Competent but not consistent handling of the laryngoscope.		Excellent handling of the laryngoscope.
Catheterisation<br>*Insert the catheter into the trachea at the desired depth and maintain the position during instillation while retracting the laryngoscope.*	Fails to insert the catheter.		Competent but not consistent handling of the catheter.		Excellent handling of the catheter.
Surfactant administration*<br>*Slow infusion to allow surfactant to be inhaled by the infant.*	Fails to administer surfactant correctly.		Competent but not consistent surfactant administration.		Excellent surfactant administration.
Complications*<br>*Manage possible complications during administration.*	Fails to manage complications.		Manages complications with some experience.		Excellent management of complications.
Ventilation*<br>*Maintain CPAP/NIV during the LISA procedure.*	Fails to maintain CPAP/NIV during the procedure.		Competent but not consistent use of CPAP/NIV during the procedure.		Excellent use of CPAP/NIV during the procedure.
Non-technical skills	*1*	*2*	*3*	*4*	*5*
Non-technical skills<br>*Situational awareness, communication, teamwork, team leadership skills.*	1<br>Fails to use non-technical skills.		Use non-technical skills with some experience.		Excellent use of non-technical skills.
Overall	*1*	*2*	*3*	*4*	*5*
Number of attempts<br>*Number of attempts until the LISA procedure is successfully performed.*	Eventually, the procedure is successfully performed.		Successfully performs the procedure by the third attempt.		Successfully performs the procedure on the first attempt.
Adherence to algorithm / Time factor*<br>*Able to follow this algorithm and minimise time delay in treatment.*	Fails to follow the algorithm.		Able to follow the algorithm but with significant time delay.		Able to follow the algorithm with minimal time delay.
Handling of the infant*<br>*Gentle handling of the patient during the LISA procedure.*	Fails to handle the patient in a gentle way.		Handles the patient with some experience.		Excellent handling of the patient.

* = Eight of the original 15 LISA-AT metrics effectively discriminated between novices and experts, equivalent to a total LISA-AT score ranging from 8-40 points.
