# Supplementary material for: Assessing competency in less invasive surfactant administration: simulation-based validity evidence for the LISA-AT scores
Source: Pediatr Res. 2025 Jan 18;98(3):876–84. doi: 10.1038/s41390-025-03868-7 (PMC12507647; doi:10.1038/s41390-025-03868-7)
Supplement: Supplementary file 2 — Supplement_Appendix_B [file 41390_2025_3868_MOESM2_ESM.pdf]

Appendix B: LISA test introduction

"*You will receive a brief introduction before you begin: You are about to perform the Less Invasive Surfactant Administration (LISA) procedure on a premature simulator. The simulator is an authentic representation of a premature infant born at 25 complete gestational weeks with a birth weight of approximately 750 grams. The infant was born one hour ago and now shows clinical signs of increased work of breathing and is receiving 35% oxygen. Normally, you would be assisted by two neonatal nurses during the procedure. I will act as both nurses, but you must tell me exactly what you want me to do. You must perform the procedure exactly as you would in a clinical setting, except here, you should verbalise your thoughts and actions so that we can assess your performance. You will perform all actions under the camera there. Would you like me to repeat the instructions? Do you have any questions before we begin?*"
