# Supplementary material for: Assessing competency in less invasive surfactant administration: simulation-based validity evidence for the LISA-AT scores
Source: Pediatr Res. 2025 Jan 18;98(3):876–84. doi: 10.1038/s41390-025-03868-7 (PMC12507647; doi:10.1038/s41390-025-03868-7)
Supplement: Supplementary file 4 — Supplement_Appendix_D [file 41390_2025_3868_MOESM4_ESM.pdf]

Appendix D

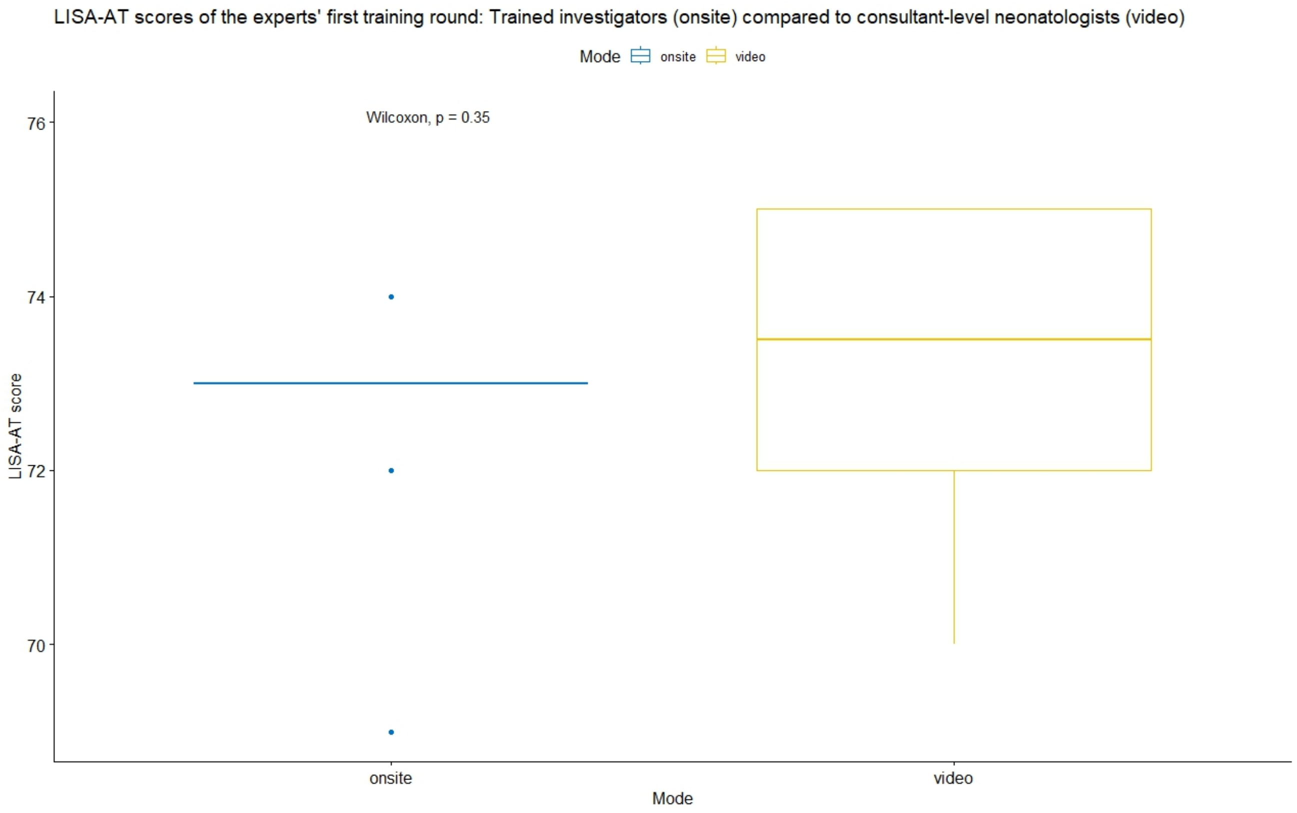

Legend: The median [IQR] of trained investigators (onsite) vs consultant-level neonatologists (video) was 73 [73-73] vs 74 [72-75], p=0.34. Therefore, the novices were trained using trained investigators (onsite) during the second training round.
