# Supplementary material for: Assessing competency in less invasive surfactant administration: simulation-based validity evidence for the LISA-AT scores
Source: Pediatr Res. 2025 Jan 18;98(3):876–84. doi: 10.1038/s41390-025-03868-7 (PMC12507647; doi:10.1038/s41390-025-03868-7)
Supplement: Supplementary file 5 — Supplement_Appendix_E [file 41390_2025_3868_MOESM5_ESM.pdf]

Appendix E

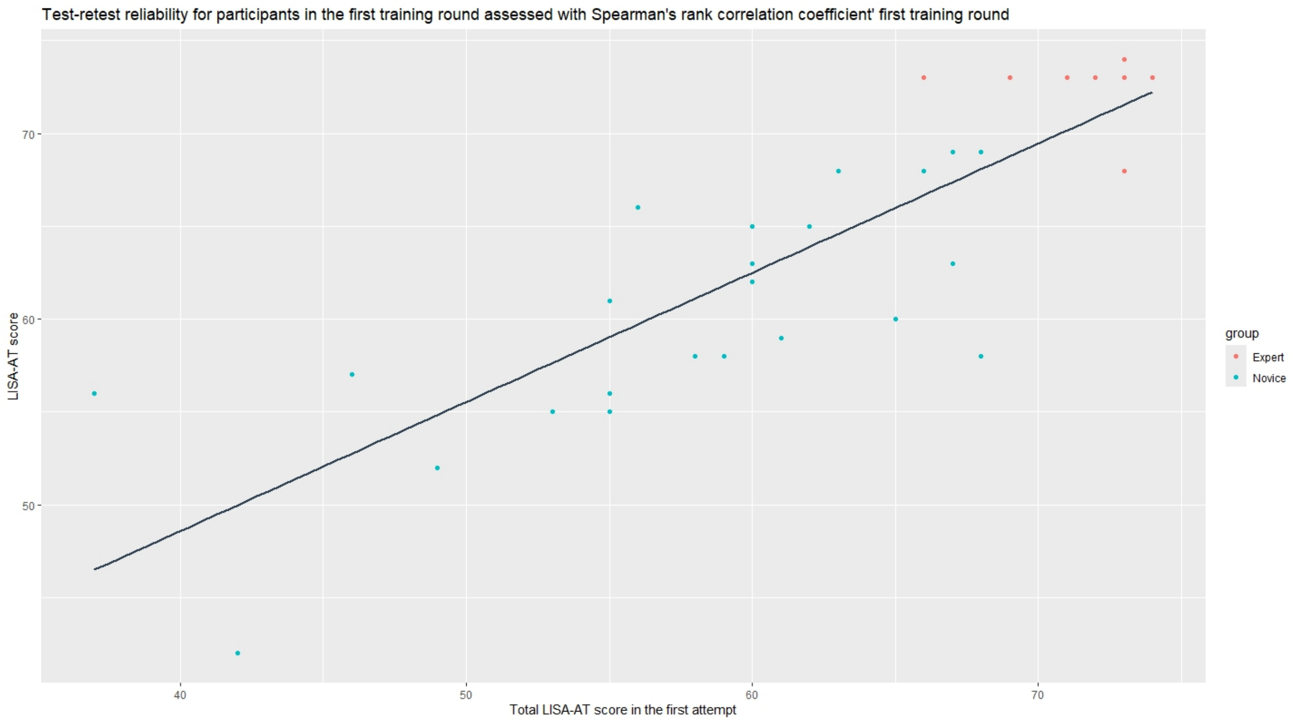

Legend: The correlation of the two performances in the first training round indicated good test-retest reliability, with a Spearman’s rho = 0.87.
