# Supplementary material for: Assessing competency in less invasive surfactant administration: simulation-based validity evidence for the LISA-AT scores
Source: Pediatr Res. 2025 Jan 18;98(3):876–84. doi: 10.1038/s41390-025-03868-7 (PMC12507647; doi:10.1038/s41390-025-03868-7)
Supplement: Supplementary file 6 — Supplement_Appendix_F [file 41390_2025_3868_MOESM6_ESM.pdf]

Appendix F

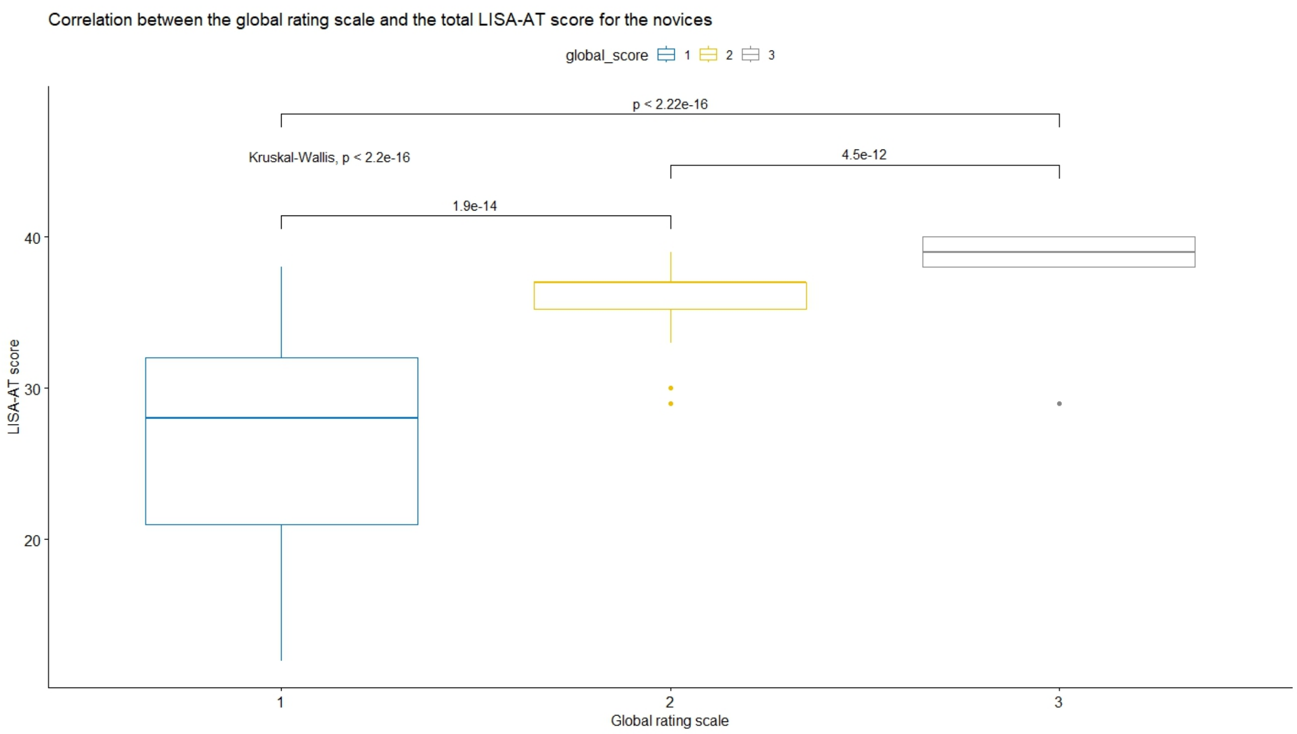

Legend: There was an overall significant correlation between the global rating scale (fail = 1, borderline = 2, fail = 3) and the total modified LISA-AT score (p<0.001). The pairwise comparisons showed significantly different distributions of the total modified LISA-AT scores within each category of the global rating scale.
