# Supplementary material for: Assessing competency in less invasive surfactant administration: simulation-based validity evidence for the LISA-AT scores
Source: Pediatr Res. 2025 Jan 18;98(3):876–84. doi: 10.1038/s41390-025-03868-7 (PMC12507647; doi:10.1038/s41390-025-03868-7)
Supplement: Supplementary file 7 — Supplement_Appendix_G [file 41390_2025_3868_MOESM7_ESM.pdf]

Appendix G

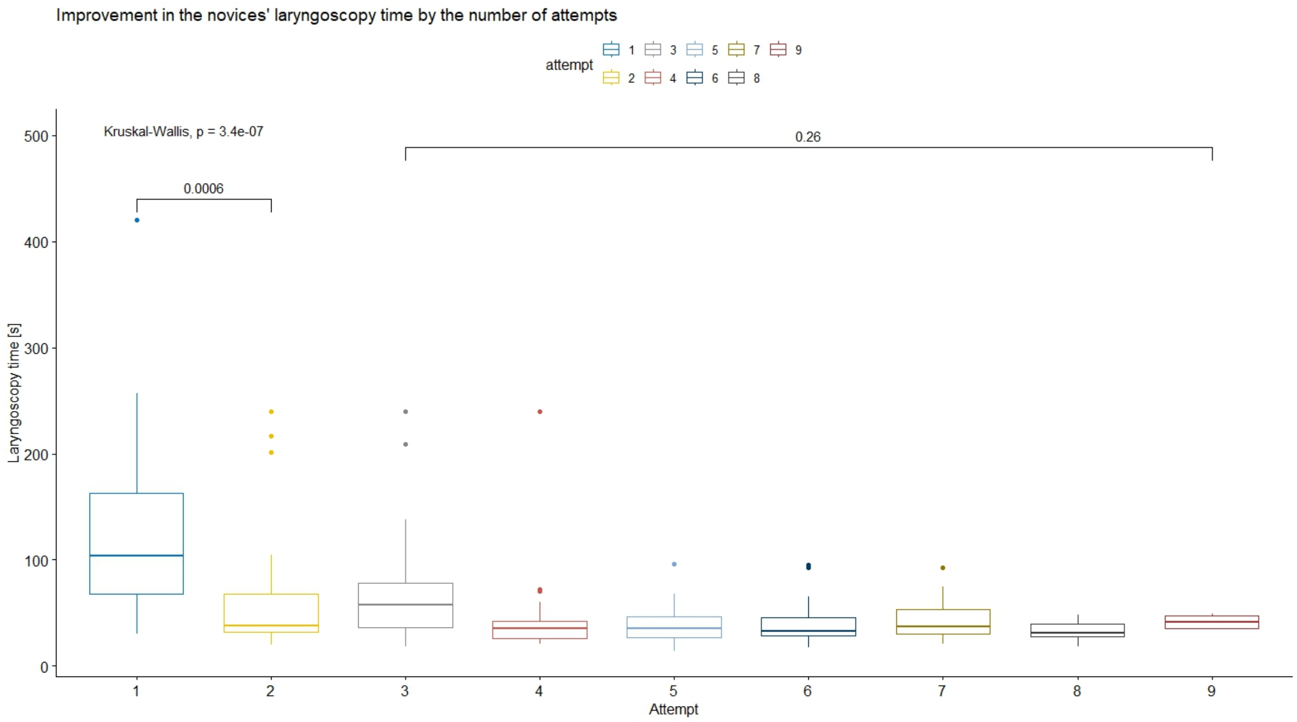

Legend: The novices' laryngoscopy time decreased significantly from the pretest to the first training round. However, there was no significant improvement in the laryngoscopy time during the second training round with feedback, which remained significantly different from the experts' performances (median [IQR] for the novices was 36 seconds [27-47] compared to 22 seconds [15-33] for the experts, p=0.001).
