# Supplementary material for: Assessing competency in less invasive surfactant administration: simulation-based validity evidence for the LISA-AT scores
Source: Pediatr Res. 2025 Jan 18;98(3):876–84. doi: 10.1038/s41390-025-03868-7 (PMC12507647; doi:10.1038/s41390-025-03868-7)
Supplement: Supplementary file 8 — Supplement_Appendix_H [file 41390_2025_3868_MOESM8_ESM.pdf]

Appendix H

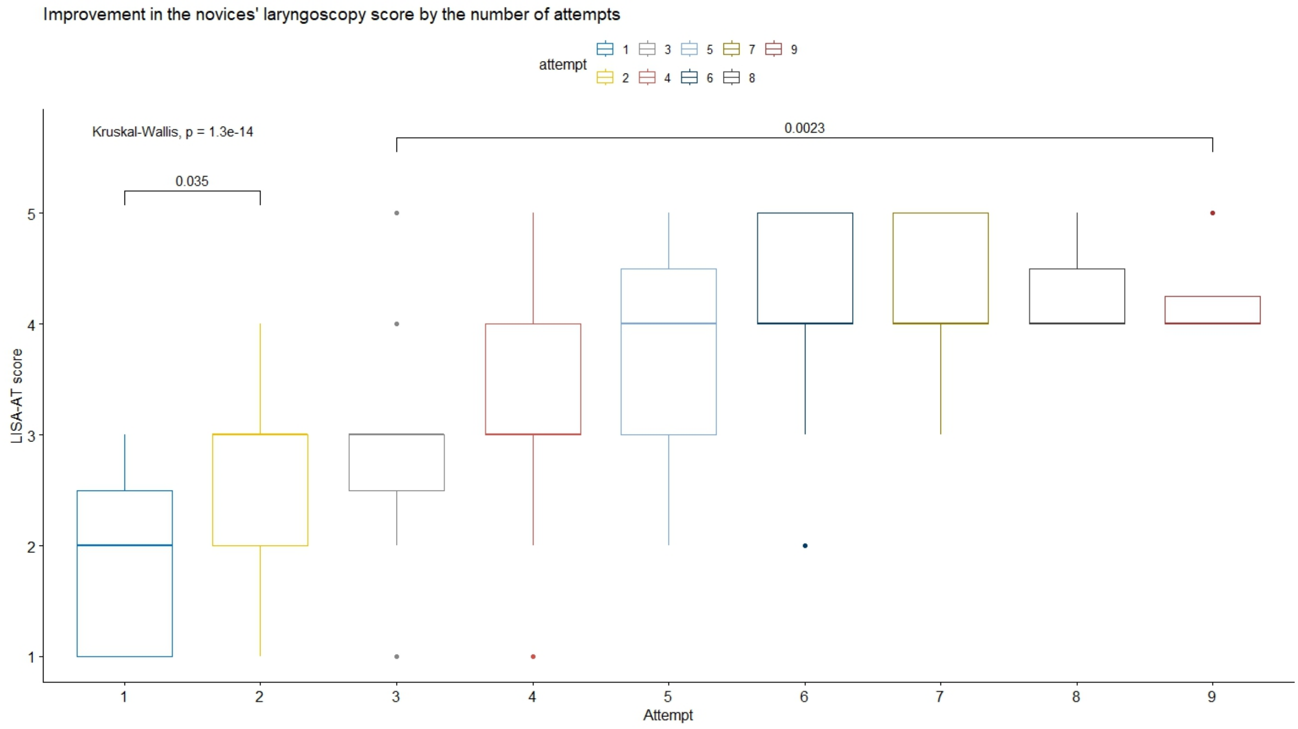

Legend: The novices' laryngoscopy skills improved significantly from the pretest to the first training round and from the first training round to the second training round. However, their laryngoscopy skills remained significantly different compared to the experts' performance (p<0.001). The "*Laryngoscopy*" metric on the modified LISA-AT was defined as: "*Competent and non-injurious handling of the laryngoscope and good visualisation of the vocal cords, including a sufficient overview of the airway*" and did not include the laryngoscopy time.
